# Opportunities for a population-based cohort in Belgium

**DOI:** 10.1186/s13690-022-00949-5

**Published:** 2022-08-11

**Authors:** Nienke Schutte, Marlies Saelaert, Petronille Bogaert, Karin De Ridder, Herman Van Oyen, Johan Van der Heyden, Brecht Devleesschauwer

**Affiliations:** 1 Lifestyle and chronic diseases, Department of Epidemiology and Public Health, Sciensano, Rue J Wytsman 14, 1050 Brussels, Belgium; 2 Department of Public Health and Primary Care, Ghent University, Ghent, Belgium; 3 Department of Translational Physiology, Infectiology and Public Health, Ghent University, Merelbeke, Belgium

**Keywords:** Health information, Cohort, Population health

## Abstract

Population-based cohorts allow providing answers to a wide range of policy-relevant research questions. In Belgium, existing cohort-like initiatives are limited by their focus on specific population groups or specific topics, or they lack a true longitudinal design. Since 2016, consultations and deliberative processes have been set up to explore the opportunities for a population-based cohort in Belgium. Through these processes, several recommendations emerged to pave the way forward – i.e., to facilitate the establishment of administrative linkages, increase digitalisation, secure long-term financial and organisational efforts, establish a consortium of the willing, and identify and tackle ethical and legal bottlenecks. This comment summarizes these recommendations, as these opportunities should be explored in depth to consolidate the existing collaborations between different stakeholders, and refers to current initiatives that can further facilitate the establishment of a Belgian population-based cohort and, more generally, administrative and health data linkage and reuse for research and policy-making.

## Introduction

The determinants of health and disease are interrelated and result from complex interactions between a wide range of environmental exposures, phenotypic characteristics and genomic factors. Moreover, public health challenges (such as the ageing population, the identification of risk factors for the development of chronic diseases or disabilities, and the assessment of the impact of health on socio-professional integration) call for new ways of using our existing health information systems. Population-based cohort studies are key in providing longitudinal data on the impact of major determinants of health, disease and disabilities and are a powerful design to capture exposure-outcome relations and explore causality [1], for example the effects of occupational, environmental and psychosocial factors on disease such as cancer or other chronic diseases, or determinants of quality of life, such as suffering from chronic diseases or environmental factors (such as pollution). In addition, the large size of population-based cohorts is required to allow for nested studies in subpopulations that still have, even in case of small effects, enough power to investigate how the interaction between health behaviour, environmental and societal factors have an impact on health outcomes. Population-based cohorts provide a sound base for targeted policy, policy follow-up and evaluation of the health and social systems.

### European cohort initiatives

Large population-based cohorts have been established in many European countries of which health, socioeconomic and occupational factors are the main focus, such as Lifelines in the Netherlands [2], HUNT in Norway [3], CONSTANCES in France [4] and the UK Biobank [5]. These cohorts are funded publicly or by a combination of public and commercial sources and include up to 500.000 participants. They comprise of longitudinal data collections by means of surveys and interviews, enriched with objective health measures including biomarkers and genomic data. Linkages are possible with various health and/or administrative registries and, in case of Lifelines, with GP records. CONSTANCES foresees yearly routine linkages whereas linkages with HUNT data are carried out on a project-by-project basis. International researchers are granted access to data and biomaterials after approval, based on scientific quality, methodology and feasibility criteria, by an appointed scientific board. Lifelines limits access to public institutes and provides data on a fee-for-service basis, whereas CONSTANCES also explicitly mentions private research groups as potential data users. Usually, additional data collections can be implemented in nested studies and HUNT even makes these data available for other researchers after an exclusive period of 4–5 years. Nested studies have already contributed to knowledge regarding, i.a., the prevalence and incidence of overweight, obesity or mental illness, the use of tobacco and electronic cigarettes, interactions between genetic variants and environmental exposures and the cost-effectiveness of population-based screenings [2–4].

Recently, the COVID-19 pandemic has demonstrated the importance of population-based cohorts for providing rapid answers to a wide range of policy-relevant research questions, such as the effectiveness of vaccination schemes [6]. Since 2016, consultations and deliberative processes have been set up to explore the opportunities for a population-based cohort in Belgium. This comment describes these developments and summarizes the main recommendations, pre-conditions and current initiatives that can pave the way towards the establishment of a Belgian population-based cohort.

### Cohort initiatives in Belgium

Belgium has some cohort-like initiatives or panels focusing on health and/or behaviour, the largest of them being part of larger European initiatives addressing specific topics, such as the Survey of Health, Ageing and Retirement in Europe (SHARE, [7]) and the Statistics on Income and Living Conditions (SILC, [8]). At national level, the Interface Demography Research Group of the Free University of Brussels (VUB) develops research activities based on a mortality follow-up of the population census 1991, 2001 and 2011. Other initiatives focus on specific subgroups of the population, such as patients (e.g. HIV cohort [9]), twins (East Flanders Twin Survey [10]), or children (Limburgs geboortecohort [11]). Additionally, the Intermutualistic Agency (IMA/AIM) governs a large population-based sample, the Echantillon Permanente Steekproef (EPS). The EPS contains a randomly sampled cohort of health insured individuals in Belgium and consists of three types of databases; demographics, reimbursed health care procedures, and reimbursed medication. Sciensano, the Belgian institute for health, is responsible for managing some of the major national (cross-sectional) health surveys, such as the Belgian Health Interview Survey (BHIS) and the Food Consumption Survey (FCS), as well as various surveillance systems.

Nevertheless, these initiatives and projects have their limitations. Some of these projects focus on specific population groups such as patients, specific occupational groups, or specific age groups. The scope of these studies is limited, based on a well-defined, specific research questions with a specific exposure and a limited number of outcome measures. Secondly, many initiatives are cross-sectional, hence leaving less opportunities to assess longitudinal exposure-outcome relations. Furthermore, some studies miss a systematic link between their research outputs and health. Especially accessing and exchanging timely health data is important for rapid response to research questions that concern current public health challenges, such as the current COVID-19 crisis. During the crisis, additional surveys were launched for example by the University of Antwerp (the Corona Study), Sciensano (COVID-19 health survey) and Ghent University (Motivation Barometer), and their results were widely used in advisory bodies and media, and presumably supported policy decisions. However, these surveys suffer significantly from self-selection. In (especially) the aftermath of this crisis, also information is needed on social, economic, cultural and physical environment in order to study the (wider) impact on population health. Yet, the rich administrative data sources in Belgium seem ‘siloed’ and the current processes of project-based linkages are complex, lack transparency and require a lot of time and resources. Also, the administrative data that are already available lack self-reported, qualitative and clinical information on the level of the person. Finally, pan-European comparative studies addressing the impact of underlying determinants of population health and providing new solutions and interventions are needed [12]. What is needed is a flexible data collection tool to facilitate data collection and structural data exchange on many topics regarding population health and social systems, facilitating research and, subsequently, decision-making processes by citizens, clinicians, public health practitioners and policy makers.

### Exploring the opportunities for a sustainable Belgian population-based cohort

In December 2016, the idea of a cohort as research infrastructure in Belgium has been discussed during a meeting with different Belgian stakeholders (universities, the Federal Science Policy (BELSPO), the Fund for Scientific Research (FWO), the National Institute for Health and Disability Insurance (RIZIV/INAMI), the Belgian statistical office Statbel, the Belgian healthcare knowledge center (KCE), the Intermutualistic Agency (IMA/AIM), and regional authorities). There appeared to be a lot of interest in this initiative and in the idea to start up a reflection on the relevance, feasibility, cost-effectiveness and expected outcomes of a Belgian population-based cohort. In 2018, the Health Working Group for the Strategic Investment Pact (established by Prime Minister Charles Michel, aiming to provide solid advice on urgent investments in Belgium) stated in their report that ‘in line with developments in other countries, Belgium will need a cohort of its population as a research infrastructure’ [13].

The Belcohort project (2018–2020) aimed to take the next step and explored the opportunities for establishing a population-based cohort. In 2019, several stakeholder meetings were organized with an international workshop on cohort studies, with key persons from population-based cohorts abroad and Belgian stakeholders representing policy makers at federal level, researchers from most Belgian universities, RIZIV/INAMI, Statbel, KCE and IMA/AIM and many other interested parties. In addition, an international workshop on cohort studies was organised, with both national and international experts.

## The way forward

Population-based cohorts are indispensable longitudinal study-designs to capture exposure-outcome relations regarding health and illness in the context of topical public health as well as socioeconomic and occupational challenges. Current cohort-like initiatives in Belgium have resulted in valuable research results but also have their limitations, including a limited scope with project-based linkages between data sources, a lack of longitudinal follow-up, difficulties in accessing and exchanging timely health and administrative data because of ‘siloed’ data sources with complex, opaque and time- and cost-intensive linkage procedures, and a lack of (international) collaborations between key stakeholders are among the most common barriers in cohort-like initiatives. In the next paragraphs, we summarize the main recommendations emerging from the Belcohort meetings and workshop and we describe some facilitating pre-conditions that can pave the way towards the establishment of a Belgian population-based cohort. Additionally, we refer to current initiatives that can further facilitate the establishment of a Belgian population-based cohort and, more generally, administrative and health data linkage and reuse for research and policy-making that emerged since the finalization of the Belcohort project.

### Facilitate the establishment of administrative linkages

Many high-quality data sources exist in Belgium, such as the mandatory health insurance data, the hospital discharge data, causes of death data, and disease registries such as the Belgian Cancer Registry. These provide valuable routine information on the health status of the Belgian population. Rich data are also collected by a variety of academic and governmental actors in health surveys, cohorts, etc. However, an integrated national health information system is lacking, and a sustainable and longitudinal linkage between data sources is missing, hampering the valorisation of these data sources [14]. For a population-based cohort, it is crucial that data that is collected in the cohort can be linked to external data sources, in order to paint a complete picture of the environment the individual is part of. In order to offer both the political system and scientists a new tool to respond to public health challenges, linking data from these different domains on an individual level is crucial. Routinely linking data will save valuable time and resources of researchers (as this process can currently take several months, if not years) and will support individuals working in policy and administration by providing accurate and timely numbers on incidence and prevalence, causes and prognosis. Therefore, future (national) surveys, such as the BHIS, should always foresee the necessary legal and ethical provisions to allow prospective routine linkages.

Different elements already seem to be in place to support routine linkages, including the existence of a unique national register number which can be used to link multiple databases, as well as the existence of technical platforms for data linkage and transfer. Nonetheless, there are very limited examples of routinely linked datasets, implying the existence of other barriers (cf. infra).

### Increase digitalization

Other possibilities for establishing a population-based cohort reside in digitalization: with more and more of our daily lives spent online, shifting from pen-and-paper questionnaires to online surveys has opened up a world of convenience for researchers and allowed them to reach a large number of people in a short period of time. A carefully designed and implemented online panel, that is consulted by means of surveys, can produce high-quality data at low marginal costs [15]. These panels can be a useful tool to collect information also beyond health issues, for example the LISS panel in the Netherlands (www.lissdata.nl). Such an internet panel can evolve into a cohort, when data collection is repeated over time. In other European countries, academic internet panels are used to collect information on lifestyle, health behaviour, diseases and expectations and experiences of health care users. In addition, the experiences of the web-based mode of the European Health Interview Survey (EHIS) showed the possibilities for employing this mode of surveying in Belgium [16]. Ultimately, this panel can mature, and additional data collection in a clinical or experimental setting can be possible. The rapid results, the flexibility (recently demonstrated by the successes of the COVID-19 health surveys, based on the BHIS [17]) and longitudinal perspective, the opportunities for scaling up of the data collection, including clinical assessments (for example by use of wearables) and enrichment of the sample through linkages with secondary data holders, make an online panel a worthy backbone for public health research.

### Secure a long-term financial and organizational effort

While the abovementioned opportunities may build a strong foundation for a population-based cohort, its actual implementation will not be possible without sufficient funding. Long-term financial and organizational efforts will be required from the start, for which opportunities should be actively explored, e.g., in the context of citizen science or research infrastructure. Currently, funding sources for scientific research are split up in smaller grants, which are awarded in competitive application processes. This results in ad hoc data linkages and an inability to allow for long-term research goals and structural data management [12]. Stable long-term funding would increase the value of longitudinal data exponentially with the number of study waves, which is the foundation for a cohort study. More support will be found if there is a push policy which promotes data accessibility. To enhance user-friendliness, the information on the design and data content should be consistently processed and made centrally available and thus centrally managed. Previous experiences show that data is requested by and shared with third parties when it is free and easily accessible [12]. Finally, organizational effort is needed to increase adherence and avoid drop-out of the participants. In other large population-based cohorts (see introduction), the communication with participants seems to be important, rather than (monetary) incentives (although generally a travel reimbursement is offered). Of course, when drop-out rates increase, new groups of participants should be included (refreshment sample).

### Establish a “consortium of the willing”

Another step towards the development of a Belgian population-based cohort is to establish a consortium of the willing that actively involves the key players that are part of the Belgian health information system, both data holders and consumers. This consortium could be an active network of key partners in academia and administrations that combines relevant research questions and methodologies, know-how and technical advances, keeps communication lines short, and builds trust and inter-institutional and inter-regional connections [12]. Moreover, national as well as regional funding opportunities could be exploited. Nevertheless, coordination across these key partners and regions is of utmost importance. A consortium of the willing should be considered as a first step in the development, governance, and support of a population-based cohort and as an efficient pooling of expertise and resources. However, after its initial development, various means should be found to further develop this consortium, for instance by including additional data sources, and to maintain it as a long-term, sustainable infrastructure. This way, the establishment of such a consortium could be in line with the establishment of a Belgian National Node, functioning as a national liaison, as is proposed in the European Joint Action on Health Information (InfAct [18]) and followed up by its successor, PHIRI (the Population Health Information Research Infrastructure (PHIRI, https://www.phiri.eu/wp4). Such a node brings together relevant national stakeholders in the country in a systematic way and facilitates discussions on core issues on health information domains [18]. Additionally, it would be mutually beneficial to participate in international cohort networks. European and international networks with a focus on population cohorts are able to create scientific capacity, spark political goodwill, and leverage international funding opportunities such as Horizon Europe. European funded projects such as SYNCHROS (www.synchros.eu) that aim to coordinate and support the synchronisation of cohorts and population surveys in Europe and worldwide, focusing on practical, methodological, ethical and legal challenges, illustrate the importance of cohorts in the European landscape.

### Identify and tackle ethical and legal bottlenecks

The establishment of routine linkages between health and administrative data in a population-based cohort might face fundamental ethical and legal bottlenecks. From a legal perspective, the GDPR considers health data as a special category of personal data whose processing is prohibited other than in exceptional circumstances, such as explicit consent by the data subject or for reasons of public interest [19]. Whereas ad hoc linkages are possible (however they often require a lot of time), the establishment of structural linkages currently lacks a legal framework [20]. Also issues regarding data access need further clarification and this in parallel with a discussion on the applicability of property rights to health data [21]. In any case, a revision of the legislation regarding health data collection, linkage and dissemination is indispensable in the development of sustainable cohorts. From an ethical perspective, population-based cohorts allow to study exposure-outcome relations which could never be studied ethically by, for instance, randomized controlled trials. Nevertheless, they raise their own ethical concerns, such as confidentiality, protecting the data subject’s rights and freedoms, and the necessity of technical and/or organizational measures to ensure data protection and ethical compliance [22]. These concerns cannot always be met easily, since, for instance, big data has made it increasingly difficult to de-personalize data in such a way that they are legally considered anonymous [23]. Moreover, ethical values such as autonomy and justice should be balanced with other considerations, such as the research purpose, in an explicit risk-benefit ratio [23, 24]. Also citizens’ role and involvement in health data reuse, for instance by obtaining informed consent or by transparent communication on research purposes or results, is an important issue that has recently been studied in an international citizen e-consultation [25]. The Belgian project AHEAD (Towards the development of a National Health Data Platform, [26]) aims to further explore these legal and ethical bottlenecks that may hamper the development of a national health data platform. To do so, it will elaborate a case study in which the data from the BHIS will be routinely and prospectively linked with administrative health datasets, in order to create a novel prospective cohort that could function as a research infrastructure providing resources and services that enable and foster research [27].

### Current national and European initiatives

Since the completion of the Belcohort project, several national and European initiatives have been launched that can further facilitate the establishment of a Belgian population-based cohort and, more generally, administrative and health data linkage and reuse for research and policy-making.

Besides identifying legal, ethical, and technical bottlenecks, the Belgian AHEAD project also actively involves partners of the Belgian health information system more closely to identify existing digital data collections, including information about procedures to obtain and exploit the data [26]. The results of this inventory will be published on a website that will serve as a research portal that makes health data collections accessible for scientific exploitation and valorisation in (population health) research. The AHEAD project will also develop practical roadmaps with possible future scenarios for and a clear framework on health data identification, accessibility, assessment and reuse in Belgium. Projects like AHEAD can be a fertile ground for the establishment and maintenance of an active network of key stakeholders that keep the idea of a population cohort on the scientific and political agenda.

Also in line with the aim of more sustainable (administrative) data linkages, the Belgian Ministry of Public Health has asked for the development of a federal Health Data Authority (HDA, [28]). This HDA should develop and implement a policy strategy regarding health (care) data and, moreover, streamline procedures for the various health databases in a GDPR-conform way. Consequently, the HDA should become the unique point of contact concerning national health (care) data to support scientific research and policy-making initiatives.

Moreover, for the period of 2019–2025, the development of a European Health Data Space (EHDS) is a priority for the European Commission [29]. The EHDS should make health data more easily accessible and exchangeable, not only for primary use, i.e., the development of a better health care system, but also for secondary use such as health related research and policy-making. The EHDS will pay particular attention to (i) data management and rules for data exchange, (ii) data quality, and (iii) data infrastructures and interoperability. Consequently, by giving full support to the EHDS, Belgium can also strengthen its own health information system; better accessibility and interoperability of data collections will aid in linking the data within the cohort to external sources. In addition, the success of a population-based cohort is dependent on the valuable scientific evidence that is generated from such a data collection; complying with European standards for data management and data exchange can increase the use of this data collection, increasing its value and relevance.

## Conclusions

There are various opportunities for Belgium to add a population-based cohort to its public health landscape. These opportunities should be explored in depth, consolidating the existing collaborations between the different stakeholders. Belgium, being a country with many authorities, services and agencies but short communication lines, might hold the right cards to build an infrastructure holding longitudinal collections of health and social data. With a population-based cohort, Belgium will be better able to provide answers to policy-relevant research questions, which, as evidenced by the COVID-19 crisis, is of utmost importance.

## Data Availability

Not applicable.

## Abbreviations

AHEAD: Towards the development of a National Health Data Platform
BCFR: Belgian Cystic Fibrosis Registry
BELSPO: Federal Science Policy
BHIS: Belgian Health Interview Survey
BNMDR: Belgian Neuromuscular Diseases Registry
DIPoH: Distributed Infrastructure on Population Health
EHIS: European Health Interview Survey
EPS: Echantillon Permanente Steekproef
ESS: European Social Survey
FCS: Food Consumption Survey
FWO: Research Foundation Flanders
HDA: Health Data Authority
IMA/AIM: Intermutualistic Agency
KCE: Belgian healthcare knowledge centre
RIZIV/INAMI: National Institute for Health and Disability Insurance
SHARE: Survey of Health, Ageing and Retirement in Europe
SILC: Statistics on Income and Living Conditions
Statbel: Statistics Belgium
VUB: Free University of Brussels
